# Effects of inorganic mercury exposure in the alveolar bone of rats: an approach of qualitative and morphological aspects

**DOI:** 10.7717/peerj.12573

**Published:** 2022-01-26

**Authors:** Paula Beatriz de Oliveira Nunes, Maria Karolina Martins Ferreira, Deborah Ribeiro Frazão, Leonardo Oliveira Bittencourt, Victória dos Santos Chemelo, Márcia Cristina Freitas Silva, Armando Lopes Pereira-Neto, Alan Rodrigo Leal Albuquerque, Simone Patricia Aranha Paz, Rômulo Simões Angélica, Sofia Pessanha, Rafael Rodrigues Lima

**Affiliations:** 1 Laboratory of Functional and Structural Biology, Institute of Biological Sciences, Federal University of Pará, Belém, Pará, Brazil; 2 School of Dentistry, Federal University of Pará, Belém, Pará, Brazil; 3 Institute of Geosciences, Federal University of Pará, Belém, Pará, Brazil; 4 Laboratory of Instrumentation, Biomedical Engineering and Radiation Physics, NOVA School of Sciences and Technology, Caparica, Portugal

**Keywords:** Inorganic mercury, Mercury chloride, Alveolar bone, Rats, Infrared mineral characterization, Micro-computed tomography

## Abstract

**Background:**

In comparison to organic mercury (MeHg), the environmental inorganic mercury (IHg) can be found in some skin-lightening cosmestics were considered “harmless” for a long time. However, recent studies have shown that long-term exposure to low doses of IHg may affect biological systems. Therefore, this study investigated the effects of IHg long-term exposure to the alveolar bone of adult rats.

**Methods:**

Adult *Wistar* rats were distributed in control and HgCl_2_ exposed (0.375 mg/kg/day). After 45 days, the rats were euthanized and both blood and hemimandibles were collected. Total blood Hg levels were measured and both inorganic and organic components of the alveolar bone were determined through XRD and ATR-FTIR. The microstructure of the alveolar bone was assessed by using micro-CT and the morphometric analysis was performed by using stereomicroscopy.

**Results:**

Alterations in the physicochemical components of the alveolar bone of exposed animals were observed. The bone changes represented a tissue reaction at the microstructural level, such as bone volume increase. However, no significant dimensional changes (bone height) were observed.

**Conclusion:**

Exposure to IHg at this dose can promote microstructural changes and alteration in the organic and inorganic components in the alveolar bone.

## Introduction

Mercury (Hg) is considered the 10th most harmful element to human health (WHO, 2017). Inorganic (elemental, inorganic salts, *e.g*.) and organic (methylmercury, dimethylmercury, *e.g*.) are the two primary Hg chemical forms, which can be bioaccumulated in the biosphere through a dynamic biogeochemical cycle (Sakamoto, Nakamura & Murata, 2018; Siciliano et al., 2005).

Anthropogenic actions such as inappropriate industrial waste management and illegal artisanal gold mining resulted in Hg contamination of ecosystems and became a public health concern. It has been shown that Hg levels in commercially available fishes may reach up to 8.71 μg/g, of which 0.3 μg/g corresponds to inorganic Hg (IHg) (Rodríguez Martín-Doimeadios et al., 2014). Moreover, the significant increase in illegal Amazon deforestation corroborates the increase of Hg emission, which results in higher environmental contamination and human exposure to Hg (Crespo-Lopez et al., 2021).

It is worthwhile to consider that humans can be daily exposed to IHg through water, seafood, vegetables, and cosmetics (Park & Zheng, 2012; Risher & De Rosa, 2007; UNEP, 2013). In comparison to organic forms, the physical-chemical characteristics of IHg limit its absorption and body distribution. Hg compounds also present two different oxidation states named mercurous (Hg^+^) and mercuric (Hg^+2^), the latter of which has a high affinity for sulfhydryl groups that favors the absorption of IHg by albumin, glutathione, amino acids, and other proteins. Thus, IHg is transported by intestinal enterocytes and renal epithelial cells, reaches the systemic circulation, and is delivered to target organs (Bridges & Zalups, 2010, 2005, 2004; Ballatori, 2002).

Toxic effects of IHg have been observed in the hematopoietic system, salivary glands, and the central nervous system (Bernhoft, 2012; Rice et al., 2014; Aragão et al., 2018; Ahmad & Mahmood, 2019; Corrêa et al., 2020); however, there is scarce evidence on IHg toxicity on bone tissue such as the reduction of mineral density in long bones (Jin et al., 2002). Alveolar bone is a mineralized connective tissue derived from the ectomesenchyme. Besides supporting teeth (Zhou et al., 2018), this tissue plays a significant role in the distribution and absorption of occlusal forces that leads to the continuous bone remodeling process (Monje et al., 2015). It must be emphasized that this tissue has different responsiveness to bone resorption and remodeling and some mechanical and biochemical stimuli can alter both the quality and quantity of alveolar bone (Tanaka et al., 2002; Mavropoulos, Rizzoli & Ammann, 2007).

Only one study recently investigated the effects of MeHg exposure on the alveolar bone (de Oliveira Lopes et al., 2021); thus, this original study aimed to investigate the effects of IHg long-term exposure on the alveolar bone of adult rats.

## Materials and Methods

### Animals and experimental groups

In this study, 20 Male Wistar rats (*Rattus norvegicus*), with 90 days old and 170–180 g, were obtained from an animal facility at the Federal University of Pará. The animals were simple randomized and allocated in collective cages with four animals per cage, where they received food and water *ad libitum*. Besides, the rats were submitted to 12 h of light/dark and maintained in a temperature-controlled room (25 ± 1 °C). All procedures were approved by the Ethics Committee on Experimental Animals of the Federal University of Pará (UFPA), under number 9228050418, according to NIH Guide for the Care and Use of Laboratory Animals recommendations and The ARRIVE guidelines (Percie du Sert et al., 2020).

**Figure 1:**
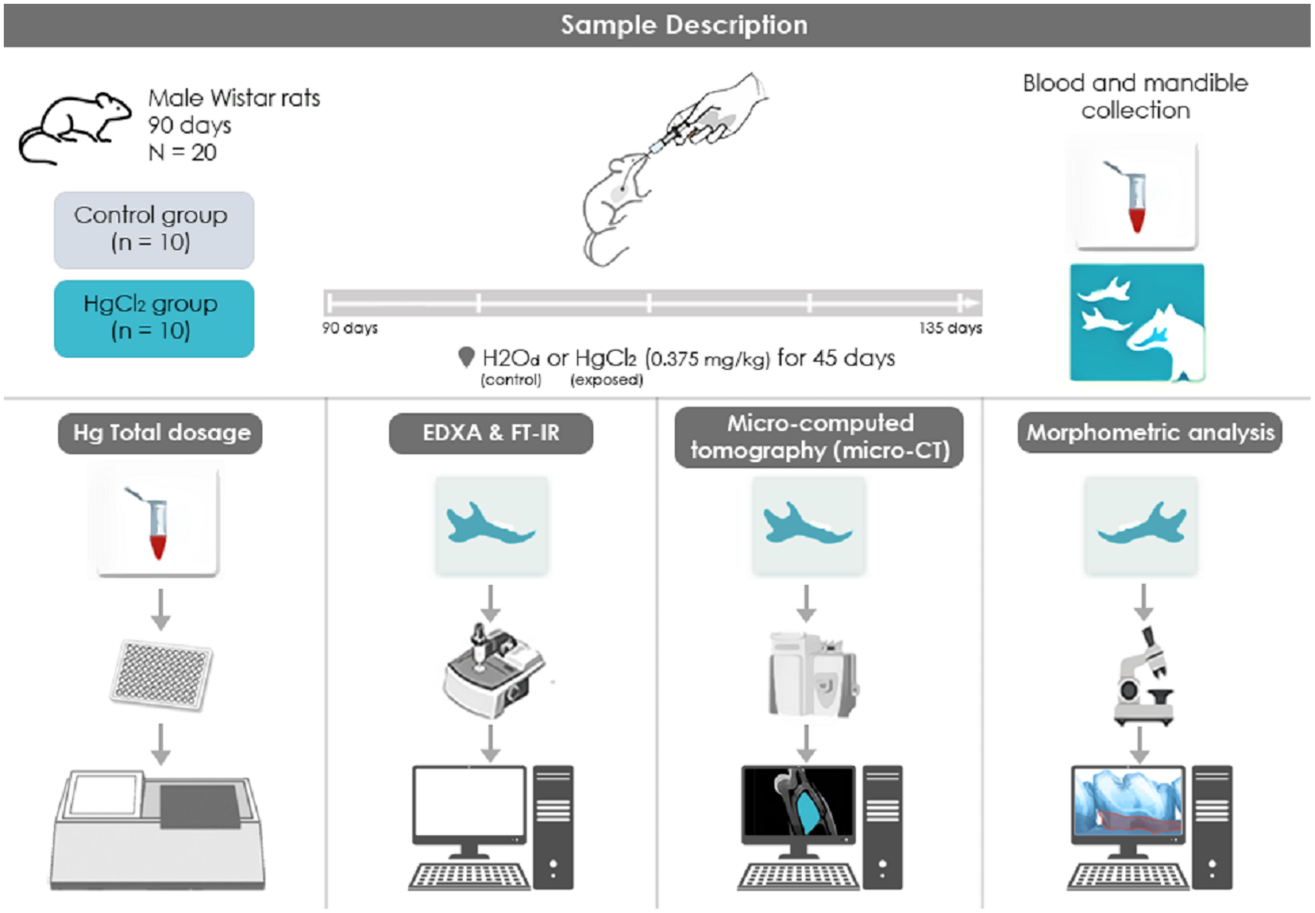
Sample description and experimental stages methodological figure of IHg exposure, experimental group division, and analyses performed. (1) HgCl_2_ exposure to 0.375 mg/kg/day by intragastric gavage for 45 days; (2) euthanasia and biological sample collection procedures (blood and mandibles); (3) total mercury levels by atomic absorption spectrometry. (4) Physicochemical analysis of the alveolar bone through Fourier Transform Infrared Spectroscopy (FT-IR); (5) Micro-computed tomography (micro-CT) analyze and (6) alveolar bone morphometric analysis by stereomicroscope.

The mercury chloride (HgCl_2_) was orally administered to the animals of this group (*n* = 10) with a concentration of 0.375 mg/kg for 45 days, adjusting the dose according to the animals’ weight. We used the protocol that was previously established by our group (Teixeira et al., 2014; Aragão et al., 2017, Aragão et al., 2018; Teixeira et al., 2018; Teixeira et al., 2019; Corrêa et al., 2020). The control group (*n = 10*) received distilled water by intragastric gavage for the same period in proportional volumes. Twenty-four hours after the last HgCl_2_ administration, the animals were euthanized with ketamine (90 mg/kg) and xylazine (10 mg/kg), and then the blood was collected for the Hg levels quantification. Also, mandibles were collected and divided for different analyses. Also, one hemimandible from each animal were maintained in saline solution (0.9%) stored in −20 °C refrigerator for mineral content analyses, while the other hemimandible was maintained in a 4% formalin solution for stereomicroscopic and microtomography analyses. For the FT-IR and XRD analyses, the alveolar bone was macerated until fine granulation powder was obtained, being then stored in different microtubes and destined for the respective analyses. All the methodological steps are summarized in Fig. 1.

### Total Hg quantification

After blood collection through intracardiac punctuare, the samples were centrifuged (3,000 rpm for 10 min) for plasma collection. Then, 1 mL of each sample was digested by an acid solution (nitric, perchloric and sulfuric, 1:1:5, v/v) and added to an electric hot plate at a temperature between 200–300 °C, and then the samples were analyzed in an atomic absorption spectrophotometer (Semi-Automatic Mercury Analyzer-Hg 201) to determine the total Hg levels, considering the detection limit 0.001 mg kg^−1^. The results were expressed in μg/mL (Dos Santos Chemelo et al., 2020).

### Crystallography analysis

The alveolar bone samples already prepared were destined for XRD analysis, which was performed using an X-Ray Diffractometer, model Empyrean from PANalytical, from the Laboratory of Mineral Characterization-X-Ray Sector. The equipment has an anode ceramic X-ray tube with Co (Kα1 = 1,789010 Å), long fine focus, Fe Kβ filter, PIXCEL3D-Medpix3 1 × 1 detector, in scanning mode. The analytics condition was with 40 kV voltage, 35 mA current, step size 0.0263° in 2θ, scan from 3.00° to 95.00° in 2θ, time/step 59.92 s, 1/8° of the divergent slit and 1/4° of anti-scattering, with 10 mm mask. Sample preparation was carried by micropreparation method using a silicon zero background sample holder.

The software X’Pert Data Collector 5.1 collected the data, and the software X’Pert HighScore Plus 4.7 made the data processing. The identification of minerals or crystalline phases was made by comparing the diffractogram obtained with standards (cards) from the ICDD-PDF (International Center for Diffraction Data–Powder Diffraction File) database.

### Infrared mineral characterization (FT-IR)

After pretreatment, samples were dried at 60 °C for 24 h and destined for FT-IR analysis were in a performed Thermo Spectrometer (Nicolet iS50) in the 4,000–400 cm^−1^ spectral range. Each spectrum is the sum of 100 scans with 4 cm^−1^ resolution. The OMNIC software was used to obtain the data. As a pre-treatment, the samples were dried at 60 °C for 24 h and analyzed in the Attenuated Total Reflection (ATR) technique which performs direct non-destructive analysis of the solids. The vibrational values established were registered as proposed by Lopes et al. (2018).

### Micro-computed tomography analysis (micro-CT)

For this analysis, the hemimandibles were digitized and the alveolar bone was evaluated in it, by a cone-beam micro-CT system (Skyscan 1172; Bruker, Kontich, Belgium). The X-ray generator was operated at an accelerated 60 kV potential, with a 165 µA beam current and a 650 ms exposure time per projection. Images were produced with a voxel (6 × 6 × 6 mm). The region of interest (ROI) was outlined from the apexes of the second molar roots up to the roof of the first molar’s furcation, touching the roots surfaces, in all images of the coronal dataset, using appropriate software (Data Viewer software, Bruker-Skyscan). Percentage of bone volume (BV/TV); trabecular spacing (Tb.Sp; mm); trabecular number (Tb.N; mm^−1^) and bone surface/volume ratio (Bs/TV) were the parameters analyzed.

### Morphometric analysis

The left hemimandible was used to measure the area between the cementoenamel junction (CEJ) and alveolar bone crest (ABC) of second molars. This measurement is considered a sensitive parameter of alveolar bone loss (Lopes Castro et al., 2020; Frazão et al., 2020). So, the hemimandibles were immersed in 8% sodium hypochlorite for 4 h, then washed in running water so that they could be immediately dried and immersed in 1% methylene blue (Sigma-Aldrich, St. Louis, MO, USA). Subsequently, we obtained the second molars’ lingual surface exposed area images with a stereomicroscope (M205A; Leica, Wetzlar, Germany) and imaging software (LAS-X, Leica). The images’ limits were the CEJ, ABC, mesial and distal surfaces of the second molars.

### Statistical Analyses

All data were inserted at GraphPad Prism 5.0 and summited to the normality test. The method of Kolmogorov–Smirnov (*p* > 0.10) tested the data distribution. For the variables with normal distribution, the Student’s *t*-test was used, considering a significant *p* < 0.05. For the body mass evaluation, the Two-way ANOVA test was applied by the Tukey test to perform a comparison between groups. The results were expressed graphically and numerically with mean and standard error (mean ± standard error) for those showing parametric.

The power of the test was analyzed, calculating the difference between two mean by OpenEpi software (versão 2.3.1) adopting type I error of 5% and power of 80% (Table S1). The number of animals required for this experiment was calculated using the OpenEpi software, with criteria for analysis of variances, based on the study in 2021 by de Oliveira Lopes et al. (2021); the α err prob was 0.05, and a power of 0.80.

## Results

### Exposure to IHg did not affect the animals’ body weight

**Figure 2:**
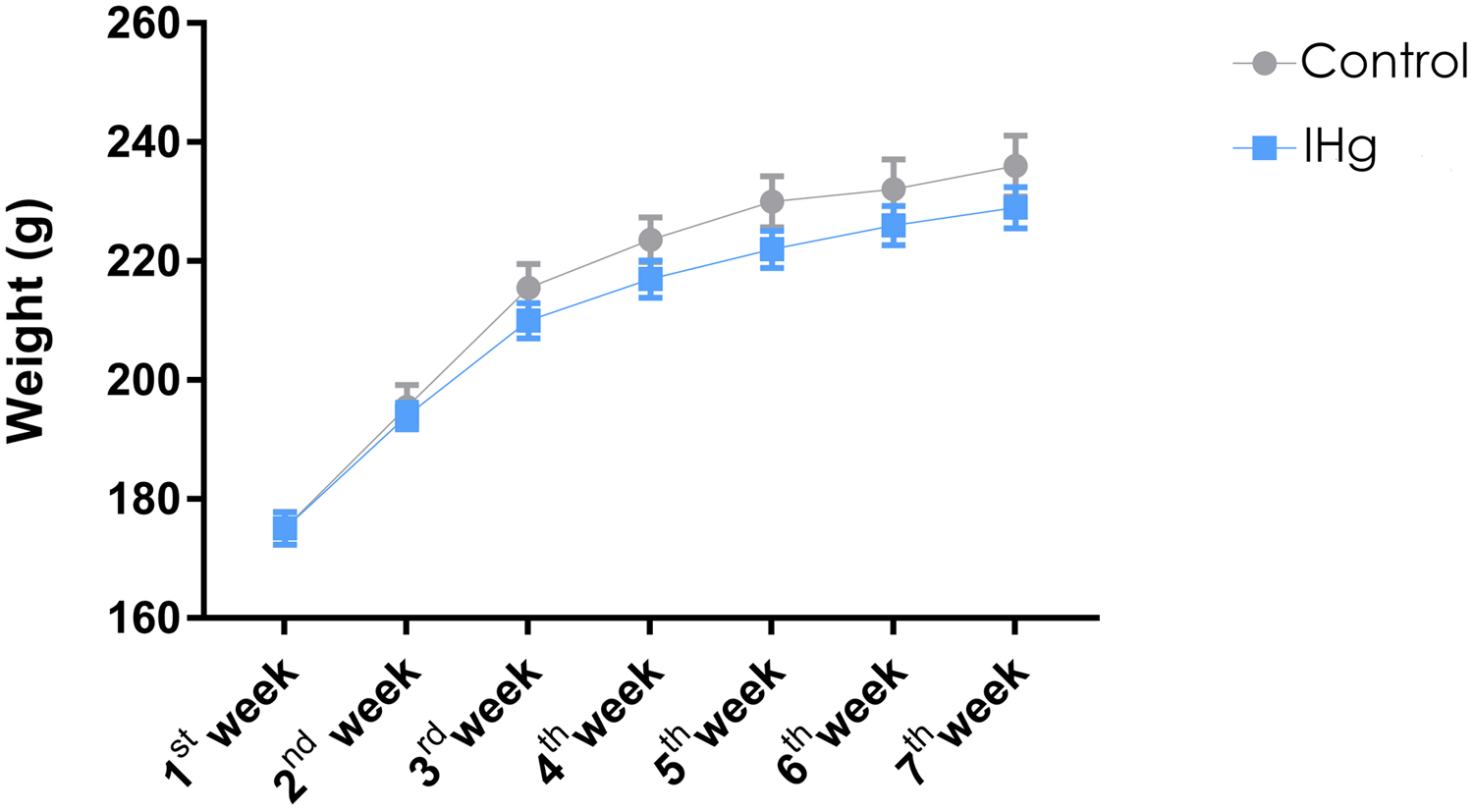
Effects of chronic exposure to IHg on body weight (g) of adult Wistar rats. Results are expressed as mean ± standard error mean. No significant differences (*p* > 0.05) between groups at any time. Two-way ANOVA and Tukey’s post-hoc test, *p* < 0.05.

The animals were weighed weekly, and the animals in both groups gained weight (*p* < 0.0001). At the end of the experimental protocol, the animals in the control group and the exposed group (IHg) showed no difference in body weight (Control: 215.4 ± 8.457; IHg: 210.4 ± 7.372, *p* > 0.05) (Fig. 2).

### The long-term exposure to IHg increases total Hg bioavailability in adult rats

**Figure 3:**
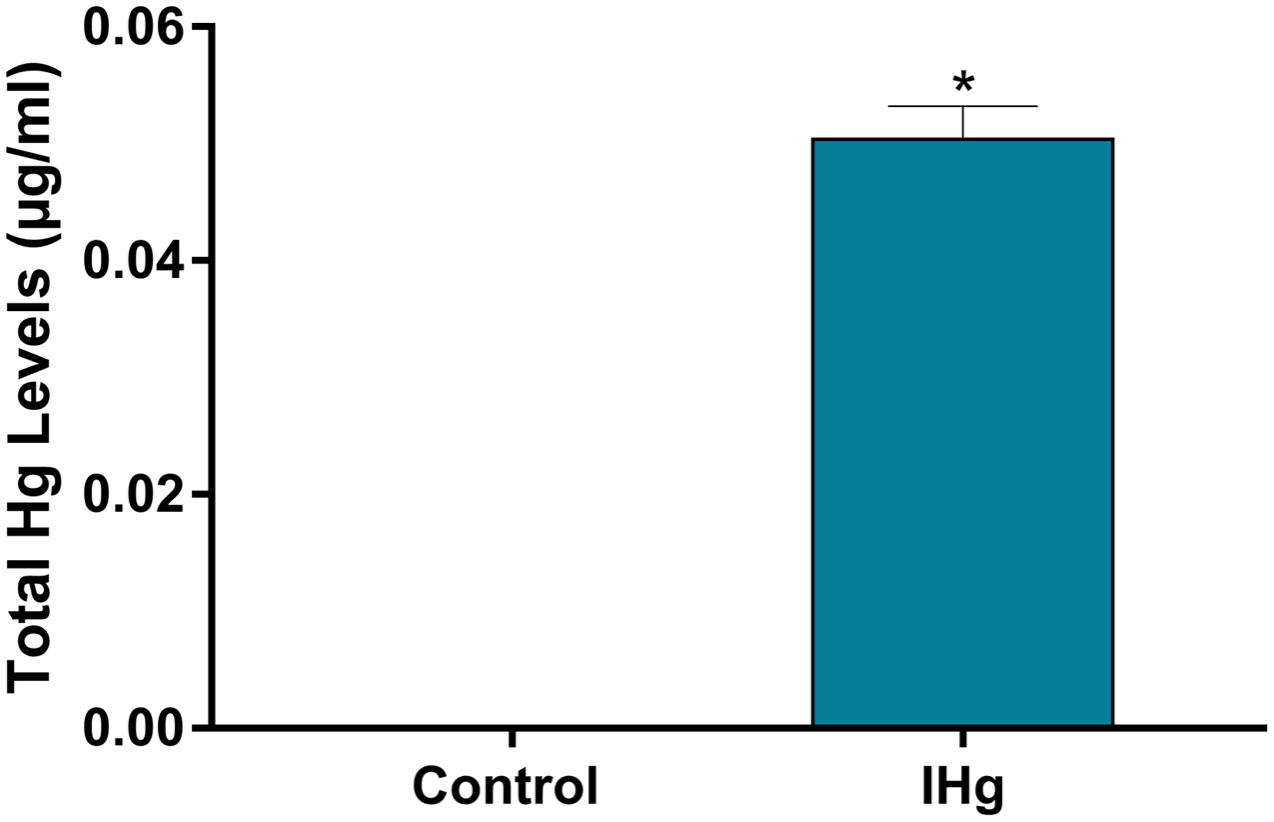
Total Hg (Hg) levels found in the plasma of adult Wistar rats after 45 days of exposure to 0.375 mg/kg/day of HgCl_2_. Results are expressed as mean ± standard error mean (SEM). *p < 0.05 compared to the control group.

As a validation of our experimental design of Hg exposure, total Hg levels in blood were higher in exposure rats (0.048 ± 0.002 µg/mL) when compared to controls (values lower than the detection limits; *p* < 0.0001) (Fig. 3).

### Changes in the parameters between groups were observed during the characterization of organic and inorganic components (FT-IR)

**Figure 4:**
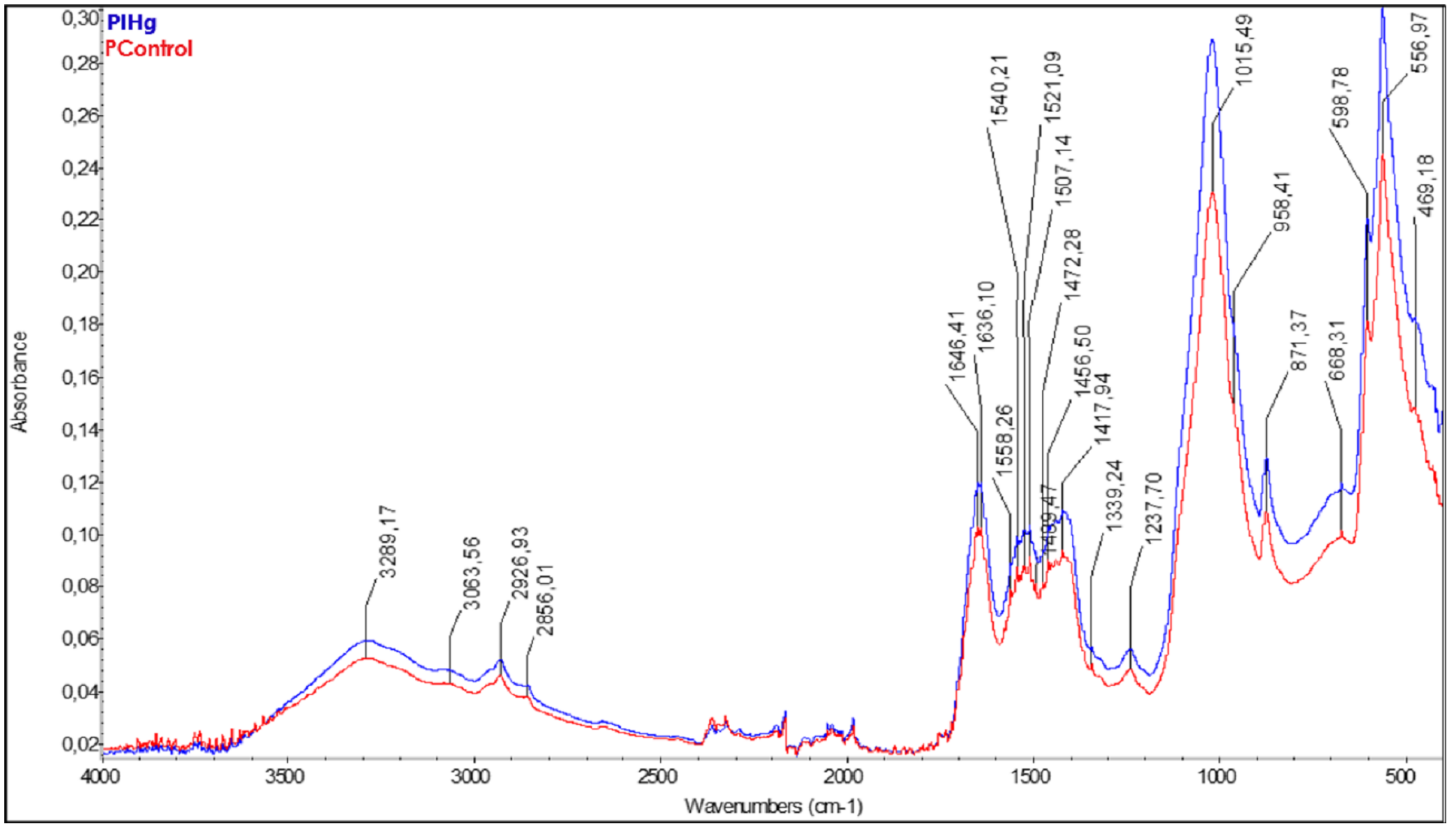
The characterization of organic and inorganic components presented changes in these parameters between groups (FT-IR). Effects of IHg exposure (0.375 mg/kg/day HgCl_2_) during 45 days on FT-IR infrared spectroscopic profile analysis of alveolar bone of adult rats (90-days-old, *n* = 10 animals/group). The qualitative results were expressed by absorbance as a function of wavelength (cm^−1^) in the comparison between FT-IR spectra of the control group (red line) with the exposed group (blue line).

The FT-IR results showed different bands between the groups exposed to IHg (blue line) and the control group (red line). The FT-IR spectrum of the rats’ alveolar bone exposed to IHg suggests modulation in the three vibrational modes: in the phosphate components (V_1_-958; V_2_- 469; V_4_-556 cm^−1^), in carbonate (V_2_-871 cm^−1^), in the amide I + H_2_O (1,646.41 cm^−1^), amide II+CO_3_ (1,540.21 cm^−1^), and amide III (1,237.70 cm^−1^). All these parameters were significant when compared to the controls (Fig. 4).

### The characterization of mineral components showed an increase in crystallinity after exposure to IHg

The characterization of mineral components by X-ray diffraction (XRD) showed a slight increase in the hydroxyapatite crystallinity in the alveolar bone after exposure to IHg. The comparison was made between the X-ray diffraction pattern of the alveolar bone of the control group (red line) with the IHg group (blue line). The vertical black lines are the peak positions of the hydroxyapatite standard (ICDD-PDF 34-0011).

**Figure 5:**
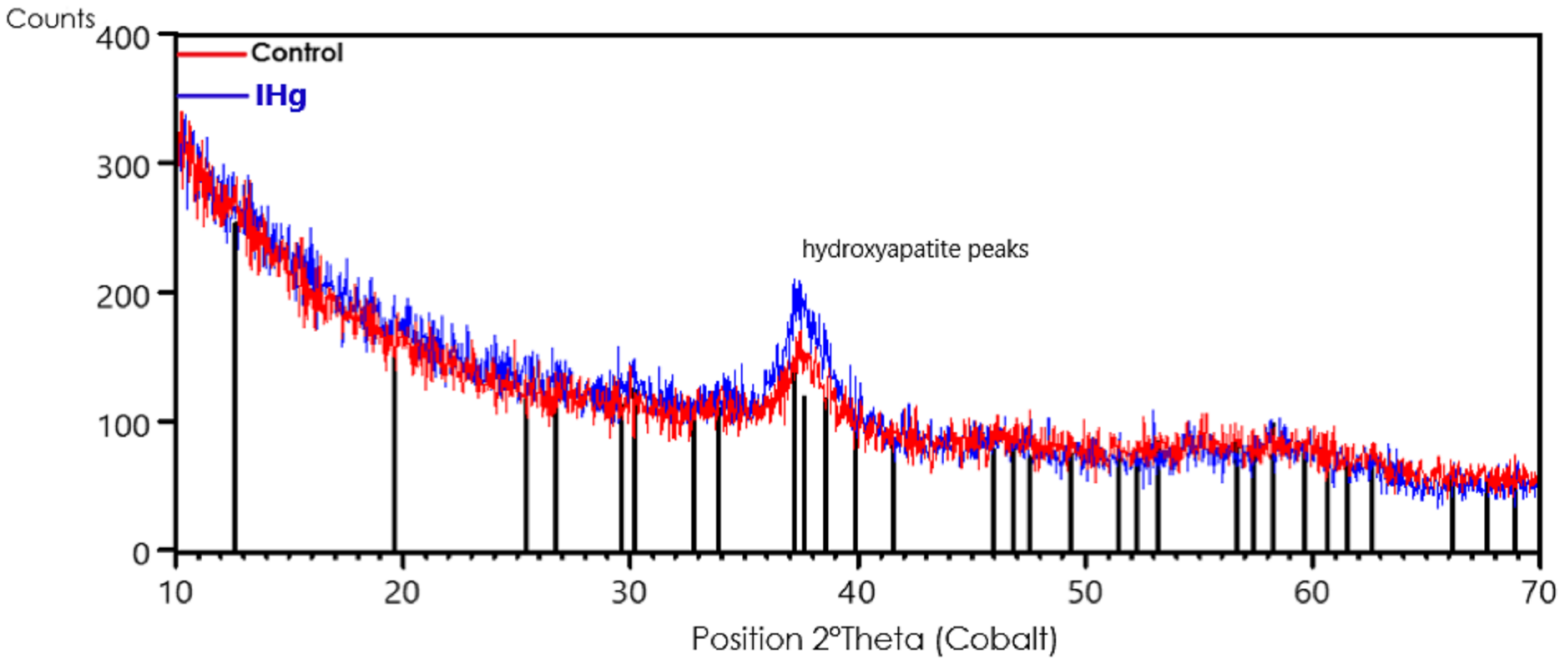
Effects of exposure to IHg (0.375 mg/kg/day HgCl_2_) for 45 days on X-ray diffraction (XRD) analysis of hydroxyapatite in alveolar bone from adult rats (90 days of age, *n* = ten animals/group). The characterization of mineral components showed an increase in crystallinity after exposure to IHg. Qualitative chemical results show the comparison between the XRD peak positions of the control group (red line) with the exposed group (blue line). The vertical black lines are the peak positions of the hydroxyapatite standard (ICDD-PDF 34-0011).

The presence of hydroxyapatite with low intensity and wide peak at 37.2° in the control group and a slightly more intense and symmetrical peak in the exposed group shows, qualitatively, an increase in the crystallinity of the rats exposed to IHg, as shown in Fig. 5.

### The exposure to IHg generated microstructural reactions in the alveolar bone

**Figure 6:**
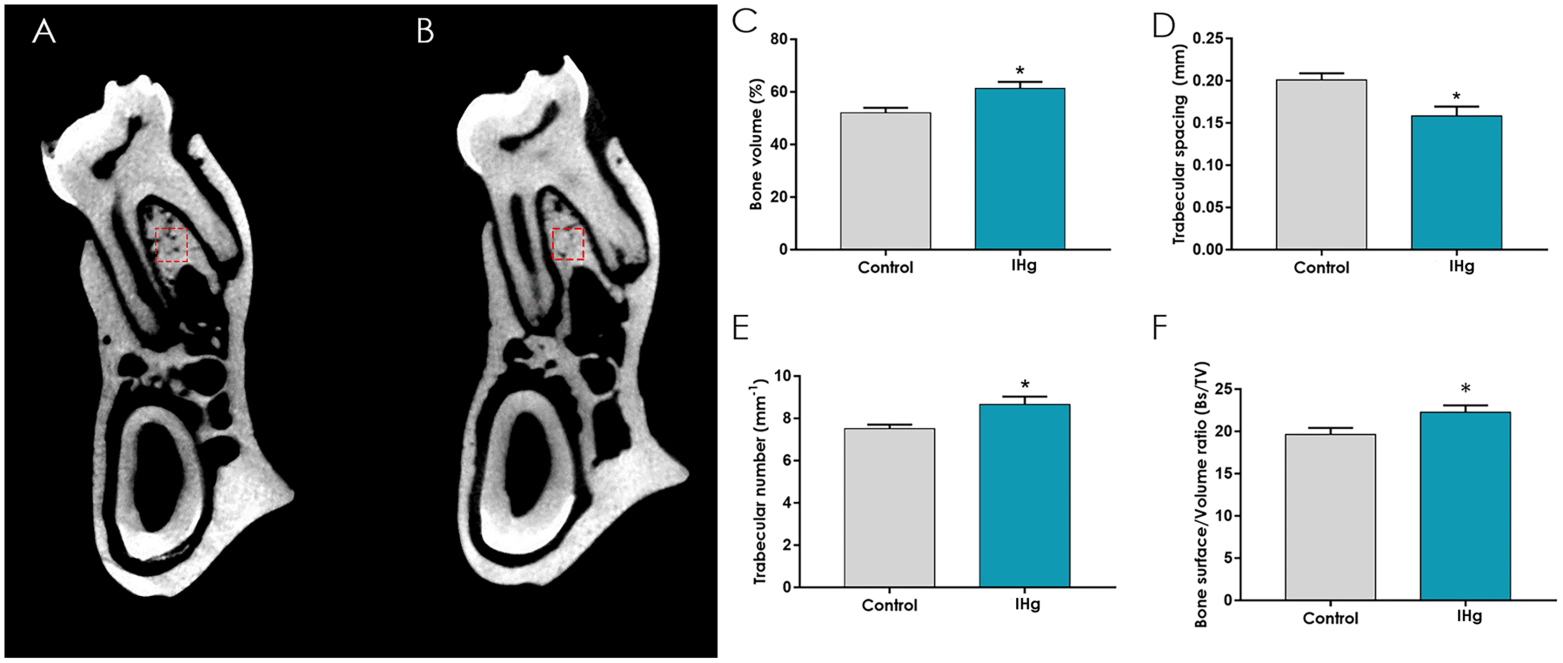
Effects of IHg exposure (0.375 mg/kg/day HgCl_2_) during 45 days on the quality of the alveolar bone of adult rats (90-days-old). (A and B) The region of interest (ROI) was represented by the interradicular region on the furcation area. Image A represents the control group and B the exposed group. The micro-CT parameters used: (C) bone volume (BV/TV; %); (D) trabecular spacing (Tb.Sp; mm); (E) trabecular number (Tb.N; mm^−1^); and (F) bone surface density (Bs/TV). Results are expressed as mean ± standard error mean. Student’s *t*-test, *p < 0.05.

The micro-CT analysis revealed that long-term exposure to IHg could cause a change in the microstructure of the alveolar bone. Hence, the IHg was able to stimulate an increase in the trabecular number when compared to the control group (IHg: 0.86 ± 0.37 mm^−1^; Control: 0.75 ± 0.19 mm^−1^, respectively, *p* = 0.04, Fig. 6E), as well as a decrease in the space between the bone trabeculae in the exposed group (IHg: 0.15 ± 0.01 mm^−1^; Control: 0.20 ± 0.007 mm^−1^, respectively, *p* = 0.01, Fig. 6D).

Moreover, the alveolar bone showed a tissue response to the toxicant, increasing bone volume/bone surface (Bv/Tv) (IHg: 61.35 ± 2.49; Control: 52.04 ± 1.95; *p* = 0.01, Fig. 6C) and one of the density parameters bone, the fraction of the bone surface/volume ratio (Bs/Tv) (IHg: 22.28 ± 0.8; Control: 19.65 ± 0.7; *p* = 0.04, Fig. 6F) in the exposed group, as illustrated in the analyzed areas represented by A and B in Fig. 6.

### Exposure to IHg for 45 days did not promote alveolar bone loss on the stereomicroscopy analysis

**Figure 7:**
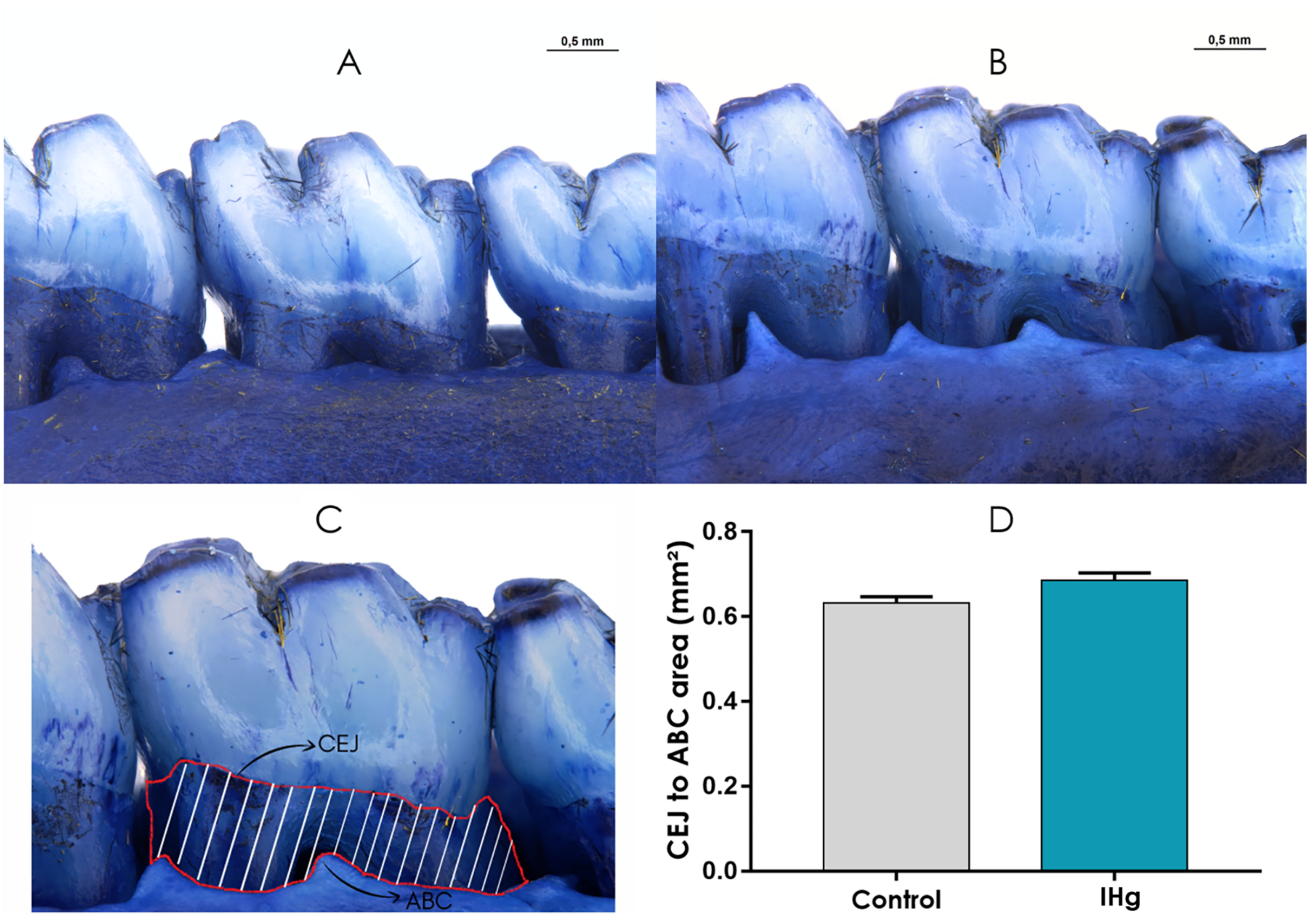
Photomicrographs of hemimandibles of the (A) control group, (B) exposed group, and (C) representative image of the exposed root area (traced in red). The graphic (D) represents the difference between the groups regarding the area between cementoenamel junction (CEJ) to alveolar bone crest (ABC) (mm^2^). Results are expressed as mean ± standard error mean. Student’s *t*-test, *p < 0.05. Scale Bar: 0.5 mm.

The stereomicroscopy revealed that the animals exposed to IHg did not show a significant difference in the exposed root area when compared to the control group (0.68 ± 0.01; 0.63 ± 0.01; *p* > 0.05) (Fig. 7).

## Discussion

This study revealed that IHg exposure altered both organic and inorganic components of the alveolar bone and increased bone mineral crystallinity. Moreover, IHg triggered a microstructural change in the alveolar bone, modulated both bone integrity and volume, and consequently decreased trabecular space. These findings suggest that the Hg levels found in the blood of the exposed animals are related to the modulation of bone microstructure; however, the reaction against Hg did not result in significant changes in the alveolar bone dimension that provides tooth support.

Several studies from our research group have shown that this IHg exposure model caused changes in blood, central nervous system, and salivary glands (Aragão et al., 2017; Aragão et al., 2018; Teixeira et al., 2018; Teixeira et al., 2019; Corrêa et al., 2020; Dos Santos Chemelo et al., 2020). This study evidenced that the oral administration of IHg can trigger changes at biochemical and molecular levels (Lohren et al., 2015; Aragão et al., 2018; Corrêa et al., 2020). Although Hg levels were not directly measured in the alveolar bone and considering the increased Hg concentrations in other tissues observed by previous studies, it can be suggested that Hg levels observed in the blood are associated with alveolar bone modulations.

It is worth mentioning that unlikely long bones, the alveolar bone has characteristics of compact bones such as fewer microporosities and a lower proportion of bone marrow (Monje et al., 2015; Zhou et al., 2018). Moreover, this tissue supports teeth that are subjected to continuous bone remodeling processes due to eruptions, occlusal forces, losses, periodontal diseases, and orthodontic movements (Tompkins, 2016). Therefore, the effects reported in long bones may differ from those observed in the alveolar bone.

Organic Hg species are predominantly reported in the literature since are widely found in contaminated fish and seafood (Rice et al., 2014). When in contact with the clouds, the Hg vapor is partially converted into IHg. Once methylated, Hg can easily cross cell membranes and is promptly absorbed into the food chain (biomagnification and bioaccumullation) (Crespo-Lopez et al., 2021; O’Connor et al., 2019). Therefore, the combination of Hg dose and the high alveolar bone turnover rate can explain the modulation of crystallinity—distribution pattern of hydroxyapatites evaluated by the characteristic spectrum of these crystals–observed in this study. The diffractograms showed that IHg exposure modulated apatite crystals, which may have increased the structural components of alveolar bone as shown by micro-CT.

The modulation of carbonate, phosphate, and amides observed through ATR-FTIR indicates that IHg exposure can modulate mineralized tissues such as alveolar bone. Some organic (amides) and inorganic (carbonate and phosphate) components are increased as a response to tissue damage and evidence a repair process (Hassumi et al., 2018). However, the increase of carbonate concentration observed in this study may be related to physical-chemical and morphological changes in mineralized tissues such as solubility increase, atomic positions variation, crystal morphology alteration, and bone mineral crystallinity decrease (Yerramshetty & Akkus, 2008; Morris & Mandair, 2011).

The literature shows that low concentrations of some metals such as zinc, copper, and nickel can play a role in bone modulation (Rodríguez & Mandalunis, 2018). Due to its morphological characteristics, the alveolar bone submitted to aggression can synthesize matrix components and increase mineralization (Zhou et al., 2018); therefore, constant bone remodeling occurs through balanced levels of RANK/RANKL/OPG and alkaline phosphatase enzymes (Cao et al., 2003; Tompkins, 2016; Hassumi et al., 2018; Page-McCaw, Ewald & Werb, 2007). The micro-CT analysis revealed that long-term IHg exposure may increase both bone volume and density, and consequently decrease the trabecular space. This study’s findings suggest that IHg was distributed throughout the body and affected the bone matrix; however, no adverse effects were observed due to protection mechanisms of the alveolar bone.

Since no significant difference in the pattern of alveolar bone loss was observed between groups, it seems that IHg can not induce horizontal and vertical loss without changing alveolar bone morphology. Thickness, height, and trabecular number are parameters that indicate the balance of the alveolar bone that support teeth (Shrout et al., 2003; Ferrare et al., 2013; Porto et al., 2020); however, the alterations observed in micro-CT and mineral composition did not compromise bone dimensions.

The stereomicroscope analysis revealed that changes in the microstructure and mineral composition of the alveolar bone did not include vertical bone dimensions. Considering that the alveolar bone vertical dimension is a relevant clinical parameter of bone health, the IHg exposure can be unnoticed for a certain period; therefore further studies with populations vulnerable to Hg exposure are encouraged.

## Conclusions

IHg exposure did not cause vertical bone loss (from the cementoenamel junction up to the alveolar bone crest), albeit modulation of physical-chemical components and microtomography parameters was observed. Despite the lack of dimension changes, the alveolar bone characterized by a high remodeling rate reacted to the toxicity of a low IHg dose.

## Supplemental Information

10.7717/peerj.12573/supp-1 Supplemental Information 1 Arrive guidelines 2.0 Checklist.

10.7717/peerj.12573/supp-2 Supplemental Information 2 Quantification of the level of total Hg in the blood, Body mass, Trabecular number (Th.N), Trabecular space (Tb.Sp), Bone volume (Bv/Tv), Bone surface/volume ratio (Bs/Tv) and bone loss of the experimental animals. Results are expressed as mean, SEM: Standard error of mean, SD: Standard deviation and Test Power (1-β error probability).

10.7717/peerj.12573/supp-3 Supplemental Information 3 Raw data.

## Additional Information and Declarations

### Data Availability

The following information was supplied regarding data availability: The raw measurements are available in the Supplemental File.
