# Supplementary material for: Effects of inorganic mercury exposure in the alveolar bone of rats: an approach of qualitative and morphological aspects
Source: PeerJ. 2022 Jan 26;10:e12573. doi: 10.7717/peerj.12573 (PMC8800384; doi:10.7717/peerj.12573)
Supplement: Supplemental Information 2 — Results are expressed as mean, SEM: Standard error of mean, SD: Standard deviation and Test Power (1-β error probability). [file peerj-10-12573-s002.docx]

| Analyze | Mean (Control) | Mean (IHg) | SEM (Control) | SEM (IHg) | Power (1-β err prob) |
| --- | --- | --- | --- | --- | --- |
| Total Hg levels | 0 | 0.048 | 0 | 0.00 (SD 0.00) | 1.000 |
| Body mass | 215.4 | 210.4 | 8.4 (SD 22.3) | 7.3 (SD 19.5) | 6.229 |
| Trabecular number (Th.N; mm^-1^) | 0.75 | 0.86 | 0.19 (SD 0.38) | 0.37 (SD 0.83) | 3.924 |
| Trabecular space (Tb.Sp; mm) | 0.20 | 0.15 | 0.00 (SD 0.01) | 0.01 (SD 0.02) | 0.9991 |
| Bone volume (Bv/Tv) | 52.04 | 61.35 | 1.95 (SD 5.1) | 2.49 (SD 6.5) | 0.8465 |
| Bone surface/volume ratio  (Bs/Tv) | 19,65 | 22,28 | 0.76 (SD 1.87) | 0.82 (SD 1.84) | 0.9986 |
| Bone loss | 0.63 | 0.68 | 0.01 (SD 0.03 ) | 0.01 (SD 0.04 ) | 1.000 |

Table 1: Quantification of the level of total Hg in the blood, Body mass, Trabecular number (Th.N), Trabecular space (Tb.Sp), Bone volume (Bv/Tv), Bone surface/volume ratio (Bs/Tv) and bone loss of the experimental animals. Results are expressed as mean, SEM: Standard error of mean, SD: Standard deviation and Test Power (1-β error probability).
